# Evaluation of Two Serological Assays for Diagnosing Zika Virus Infection

**DOI:** 10.3390/diagnostics11091696

**Published:** 2021-09-17

**Authors:** Steev Loyola, Alfredo Huaman, Dina Popuche, Elizabeth Castillo, Julia S. Ampuero, Maria Silva, Carolina Guevara, Douglas M. Watts

**Affiliations:** 1 U.S. Naval Medical Research Unit No. 6 (NAMRU-6), Lima 07006, Peru; alfredo.a.huaman.ln@mail.mil (A.H.); dina.e.popuche.ln@mail.mil (D.P.); elizabeth.l.castillo4.ln@mail.mil (E.C.); julia.s.ampuero.ln@mail.mil (J.S.A.); maria.e.silva19.ln@mail.mil (M.S.); 2 Vysnova Partners Inc., Landover, Hyattsville, MD 20785, USA; 3 Department of Biological Sciences, University of Texas at El Paso, El Paso, TX 79968, USA; dwatts2@utep.edu

**Keywords:** Zika virus, enzyme-linked immunosorbent assay, neutralization tests

## Abstract

Zika virus (ZIKV) emerged and spread rapidly in South American countries during 2015. Efforts to diagnose ZIKV infection using serological tools were challenging in dengue-endemic areas because of antigenic similarities between both viruses. Here, we assessed the performance of an in-house developed IgM antibody capture enzyme-linked immunosorbent assay (MAC-ELISA) and the plaque reduction neutralization test (PRNT) to diagnose ZIKV infection. Acute and convalescent paired serum samples from 51 patients who presented with clinical symptoms suggestive of an arbovirus illness in dengue-endemic areas of Honduras, Venezuela, Colombia and Peru were used in the assessment. Samples were tested for ZIKV, dengue and chikungunya virus using a variety of laboratory techniques. The results for the ZIKV-RNA screening and seroconversion detected by the microneutralization test were used to construct a composite reference standard. The overall sensitivity and specificity for the MAC-ELISA were 93.5% and 100.0%, respectively. Contrastingly, the overall sensitivity and specificity for the PRNT were 96.8% and 95.0%, respectively. Restricting the analysis according to IgM or neutralizing antibodies against dengue, the performances of both serological assays were adequate. The findings of this study reveal that the MAC-ELISA and PRNT would provide initial reliable laboratory diagnostic assays for ZIKV infection in dengue-endemic areas.

## 1. Introduction

In 2015, an outbreak of Zika virus (ZIKV) was reported in northeast Brazil, where other arboviruses, including dengue (DENV) and chikungunya (CHIKV) viruses, were co-circulating. Initially, the Brazilian cases were presumptively diagnosed as acute viral illnesses and were treated for a dengue-like illness. Subsequently, cases caused by ZIKV were confirmed using molecular techniques [1,2]. After the emergence of ZIKV in Brazil and its laboratory confirmation, the virus spread rapidly throughout the Americas, including Colombia, Peru, Honduras, Venezuela and other countries [3,4,5].

ZIKV is mainly transmitted by *Aedes* species mosquito but can be also transmitted through sexual contact, blood transfusion and vertically to the fetus [5,6,7]. The clinical manifestations of ZIKV infections vary from asymptomatic to severe neurologic and ocular disorders [8,9]. However, the majority of ZIKV infections are asymptomatic or cause mild to moderate illness [5,10]. Due to the high risk of developing severity among immunosuppressed, pregnant women and people with underlying medical conditions, there is a necessity for reliable and highly specific tools to diagnosed ZIKV diseases.

The diagnosis of ZIKV infection is based on the real-time quantitative reverse transcriptase polymerase chain reaction (qRT-PCR) that targets the viral RNA and serological assays that detect the immune response against the virus [11,12]. The real-time qRT-PCR is widely used during the first week of illness to test sera samples for viral RNA, and capture enzyme-linked immunosorbent assay (MAC-ELISA) is used to test serum samples for the IgM antibody collected about one week after the onset of symptoms. As a more specific assay, the plaque reduction neutralization test (PRNT) or the microneutralization test (MNT) is used to detect the virus type-specific antibody to identify the cause of a past infection or to resolve inconclusive results and confirm cross-reactive IgM antibody results [5,12,13].

An accurate diagnosis of ZIKV infection using serologic-based tests in dengue-endemic areas poses several challenges due to flavivirus cross-reactive antibodies [14,15,16]. The MAC-ELISA is reliable for detecting the virus type-specific antibody in areas where only one flavivirus is endemic, but it does not always distinguish among antibodies elicited by ZIKV and DENV in the same individual. As a result, a positive MAC-ELISA result in flavivirus-endemic areas cannot be considered confirmatory and requires further testing using highly specific tests such as PRNT [5,12,13,17]. Although these serologic techniques are essential for making an accurate serological diagnosis of ZIKV infection, they are not always available, especially in resource-limited laboratories with no access to commercial serologic tests for ZIKV antibodies. Therefore, the aim of the study was to assess the diagnostic performance of MAC-ELISA and PRNT, which were developed in-house for the diagnosis of ZIKV infection using clinical samples collected from patients who lived in flavivirus-endemic areas.

## 2. Materials and Methods

### 2.1. Dengue and Zika Viruses

The DENV used in this study included DENV-1 (strain 16007) and DENV-2 (16681) strains that were isolated from patients during 1964 in Thailand [18], DENV-3 (IQT1728) isolated in 2001 from a Peruvian dengue fever case [19] and DENV-4 (strain 1036) isolated from a patient during 1967 in Indonesia [18]. The ZIKV used in this study was isolated in 2018 from a Peruvian case. All of the viruses were used to prepare working stock viruses in African green monkey (*Chlorocebus* species) kidney epithelial (Vero-76) cells (ATCC: CRL-1587, American Type Culture Collection, Manassas, VA, USA) to perform the PRNT for testing sera samples for ZIKV and DENV antibodies.

Additionally, a ZIKV in-house antigen was prepared for use in the MAC-ELISA. The supernatant of ZIKV-infected Vero-76 cells was collected when cells displayed >75% of cytopathic effect and inactivated using 3 mM of binary ethylenimine (Sigma-Aldrich, St. Louis, MO, USA) during one hour at 37 °C followed by an incubation of 10 days at 4 °C. The working stock of ZIKV was clarified at 150 g for 10 min, and the supernatant was collected and used as the ZIKV antigen. For stability and preservation purposes, 4% of bovine serum albumin (Merck, Darmstadt, Germany) and 0.1% of sodium azide (Merck, Darmstadt, Germany) were added to all stock virus for store at 4 °C.

### 2.2. Study Population and Clinical Samples

Serum samples were collected as part of an acute febrile disease surveillance study from patients who presented with clinical symptoms suggestive of arboviral illnesses in dengue-endemic areas of Honduras, Venezuela, Colombia and Peru. The surveillance protocol was approved by the NAMRU-6 Institutional Review Board (IRB), Peru; Unidad de Investigación Científica IRB #1, Tegucigalpa, Honduras; Centro de Investigaciones Biomedicas-Universidad de Carabobo IRB, Aragua, Venezuela; and Universidad de Cartagena IRB, Colombia.

A total of 473 patients with acute and convalescent paired serum samples were enrolled from June to December 2016. Of them, 37 (7.8%) patients were positive for ZIKV only by a multiplex real-time qRT-PCR [11] using their acute serum samples. For the evaluation described here, 27 and 24 patients that tested positive for ZIKV and negative for ZIKV, DENV and CHIKV, respectively, were included. The fifty-one enrolled patients were from Honduras (*n =* 2), Venezuela (*n =* 2), Colombia (*n =* 3) and Peru (*n =* 44), and all provided authorization to use their samples for future research. These four countries reported confirmed cases of ZIKV after the outbreak in Brazil during 2015 [3,4,5]. Acute samples were collected during the first five days’ post-onset of symptoms, and convalescent samples were collected after 18.1 ± 6.1 (mean ± standard deviation) days.

All of the acute samples were simultaneously tested for ZIKV, DENV and CHIKV using a multiplex real-time qRT-PCR [11]. Paired sera samples were heat-inactivated for 30 min at 56 °C prior to serological testing. The samples were screened for IgM antibodies to ZIKV and DENV by the MAC-ELISA, and all positive samples were tested for neutralizing antibody by the PRNT. Additionally, any seroconversions for ZIKV infection were further assessed using the paired serum samples by a microneutralization test (MNT). DENV- and ZIKV-antibody-positive and negative human sera samples were included as controls in each serological test run.

### 2.3. Viral RNA Molecular Detection

Viral RNA was extracted from all 51 acute human serum samples using the QIAamp viral RNA minikit (Qiagen, Hilden, Germany), as recommended by the manufacturer. The RNA samples were tested for ZIKV, DENV and CHIKV by a multiplex real-time qRT-PCR [11]. A positive result was defined if the cycle threshold (Ct) value was ≤38 cycles, as previously described [11]. Positive, negative and no template controls for each virus were included in each plate run.

### 2.4. ZIKV MNT

Serial 2-fold dilutions of the heat-inactivated acute and convalescent sera samples ranging from 1/10 to 1/640 were prepared in 96-well flat bottom plates. A dose of ZIKV to yield 300 PFU/mL in 50 µL was added to each well that contained 50 µL of the sera dilutions. The plate was incubated for 1 h at 37 °C, 5% CO_2_. Then, 100 µL of Vero-76 cells at a density of 2 × 10^5^ in Eagle’s Minimum Essential Medium (E-MEM; Merck, Darmstadt, Germany) and 10% fetal bovine serum (FBS; Merck, De Soto, KS, USA) was added to each well and incubated for 4 days at 37 °C in an incubator with 5% CO_2_. Viable cells were stained for 1 h at room temperature using 0.1 mL/well of color stain, which was a mix of anhydrous sodium acetate (Merck, Darmstadt, Germany), naphthol blue black (Merck, Darmstadt, Germany) and acetic acid (Merck, Darmstadt, Germany). Plaque-forming units (PFU) were counted to determine the dilution of the sera samples that reduced the viral dose by 50% as an estimate of the antibody titer.

In previous experiments involving the use of the MNT in our laboratory, we did not observe cross-reaction among the 4 DENV serotypes in a set of well-characterized DENV-positive samples (Supplementary Table S1).

### 2.5. ZIKV PRNT

The PRNT for ZIKV was performed as previously described before for DENV [20]. Briefly, heat-inactivated convalescent sera samples were diluted using E-MEM 2% FBS in a 2-fold serial dilution from 1/10 to 1/320 and were placed in a 12-well plate. One hundred microliters of each serial dilution were mixed with the same volume of ZIKV containing 700 PFU/mL and then incubated for 1 h at 37 °C. Next, one mL of a suspension of Vero-76 cells at a density of 2 × 10^5^ was added to each well of 12-well plates and incubated for 30 min at 37 °C with 5% CO_2_. A 100 µL volume of each of the co-incubated sera sample-virus mixture was transferred to each of the 2 wells of cells and then incubated for 3 h at 37 °C, 5% CO_2_. Then, 1 mL of semisolid media (E-MEM 3% of carboxymethyl cellulose (Merck, Darmstadt, Germany)) was added to each well and incubated for 5 days at 37 °C, 5% CO_2_. Cells in each well were stained for 1 h at room temperature with 1 mL of color stain. The dilution of each sera sample that reduced the virus dose by 80% was recorded as the plaque reduction neutralization titer [20].

### 2.6. ZIKV MAC-ELISA

All sera samples were tested for ZIKV IgM antibody using a modified method that was previously described [21]. Briefly, a 96-well round bottom plate was coated with 50 µL of goat F(ab’)2 anti-Human IgM antibody (Jackson ImmunoResearch, West Grove, PA, USA) diluted 1:1200 with phosphate-buffered saline (PBS; Merck, Darmstadt, Germany) pH 7.4 and incubated at 4 °C overnight. Then, the plate was washed five times using 300 µL of wash buffer (PBS + 0.1% Tween-20 (Sigma-Aldrich, St. Louis, MO, USA)). Heat-inactivated acute and convalescent sera samples were diluted 1/400 in PBS, then 50 µL of this dilution were added in duplicates to each well and incubated for 1 h at 37 °C. The ZIKV in-house antigen was diluted in 1/10 in PBS with 5.0% skim milk (Merck, Darmstadt, Germany), 0.1% Tween-20 and 2% normal human serum (Invitrogen, Waltham, MA, USA). The normal human serum was negative for arboviruses including ZIKV, DENV, Yellow fever virus and Chikungunya virus. Fifty microliters of the diluted antigen were added to each well followed by an incubation for 2 h at 37 °C. Then, 50 µL of MoAb 4G2 (Merck, Darmstadt, Germany) diluted 1/1000 in PBS was added to each well and incubated for 1 h at 37 °C. Fifty microliters of goat anti-mouse IgG + M conjugated with HRP was diluted 1/4000 (Invitrogen, Waltham, MA, USA) and added to each well and incubated for 1 h at 37 °C. Unbound antibodies were removed by washing the plate three times after each step using 300 µL of wash buffer (PBS + 0.1% Tween-20). Finally, 50 µL of ABTS peroxidase substrate (KPL, Milford, MA, USA) was added to each well and incubated for 30 min at 37 °C. ZIKV-antibody-positive and negative control sera were included in each plate. The plate was read at 405/630 nm and optical density (OD) values were recorded. The cut-off was established at 0.214 by a non-parametric receiver operating characteristic analysis using the composite reference as the gold standard (Supplementary Table S2).

### 2.7. DENV MAC-ELISA

The DENV MAC-ELISA was performed as described above for the ZIKV MAC-ELISA but included minor modifications. Heat-inactivated convalescent samples were diluted 1/100, and the viral antigen used consisted of the four distinct serotypes of dengue. An in-house hyperimmune mouse ascitic fluid was produced, evaluated and titrated as previously described [22,23], and then it was used 1/1000 against viral antigens. The final volume in all steps was set at 100 µL and the incubation time at 1 h with the exception of the ABTS peroxidase substrate step, which remained as described above. Positive and negative controls DENV IgM sera samples were included in each plate. The plate was read at 405/630 nm and the cut-off was defined as previously described [21].

### 2.8. DENV PRNT

The DENV PRNT was performed in accordance to a previously described method [20]. Briefly, heat-inactivated convalescent sera samples were diluted 2-fold from 1/20 to 1/320. The four dengue virus serotypes were mixed using same volumes resulting in 100 µL and then incubated for 3 h at 37 °C, 5% CO_2_. A volume of 0.5 mL of BHK-21 CL15 cells (ATCC: CCL-10, American Type Culture Collection, Manassas, VA, USA) at a density of 3 × 10^5^ cells/mL was added to each well that contains the virus and diluted sera samples, and incubated for 30 min at 37 °C, 5% CO_2_. Finally, 0.5 mL of semisolid media was added to each well and incubated at 37 °C, 5% CO_2_ for 7 days for DENV-1 and DENV-3, and 5 days for DENV-2 and 6 days for DENV-4. Cells in each well were stained for 1 h at room temperature with 1 mL of color stain. The dilution of each sera sample that reduced the virus dose by 70% was recorded as the plaque reduction neutralization titer.

### 2.9. Data Analysis

The construction of a composite reference standard based on two or more imperfect tests reduced the bias of possible misclassification [24,25,26,27]. Here, we constructed a composite reference to classify participants based on their laboratory results. To be classified as a ZIKV-positive case, the participant had to meet at least one of the following criteria: acute sera sample tested positive for ZIKV-RNA, or a seroconversion of ZIKV infection by the MNT using paired sera samples (negative to positive, or positive to positive with a titer increase of ≥4-fold). The composite reference was used as the gold standard to determine the performance of both MAC-ELISA and PRNT for ZIKV.

We estimated the diagnostic sensitivity and specificity, as well as the percentage of correctly classified participants, to determine the optimal cut-off for the ZIKV MAC-ELISA using the receiver operating characteristic analysis (Supplementary Table S2). The overall and stratified sensitivity and specificity, as well as their 95% confidence intervals (95% CI) were calculated for the MAC-ELISA and PRNT for ZIKV. The Mann–Whitney U test was used to analyze differences in the ZIKV MAC-ELISA OD values according to the composite reference and results for the DENV MAC-ELISA. The DENV PRNT profile was defined as naïve (no previous infection), monotypic (titers for one DENV serotype or predominantly for one serotype (5 times higher)) and multitypic (infection with >1 serotype with non-predominant serotype) [28]. The agreement and correlation of PRNT and MNT results in convalescent samples was evaluated using the Kappa statistic and the Spearman’s rank correlation coefficient, respectively. The data analysis was conducted in Stata v16.0 (StataCorp. 2019. Stata Statistical Software: Release 16. College Station, TX: StataCorp LLC.) considering a *p* < 0.05 as significant.

## 3. Results

A total of 51 participants and their acute and convalescent paired samples were included in this study. According to the composite reference, 31 and 20 participants were classified as ZIKV-positive and ZIKV-negative cases, respectively (Table 1). Among the Zika cases, 26 (83.9%) were positive by the real-time qRT-PCR assay (Ct values range: 20.3 to 36.7) and all also seroconverted, 1 (3.2%) was positive by only the real-time qRT-PCR assay (Ct = 23.8), and 4 (12.9%) only seroconverted by MNT. None of the acute samples were positive for DENV-RNA or CHIKV-RNA by the real-time qRT-PCR assay.

### 3.1. Assessment of the ZIKV MAC-ELISA

**Figure 1:**
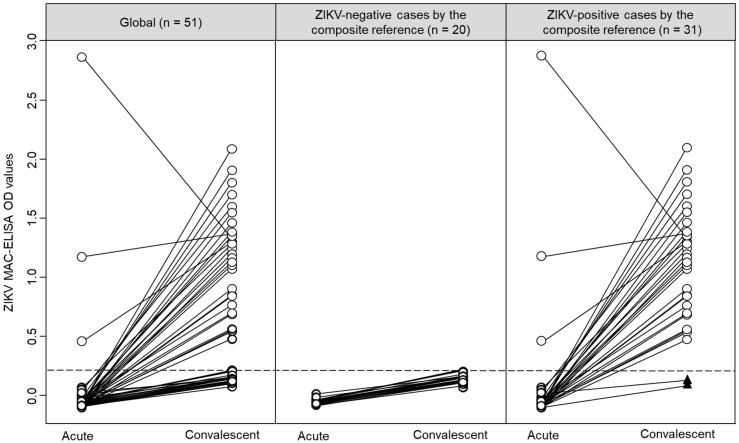
Graphical representation of the ZIKV MAC-ELISA results in paired samples, stratified by the composite reference. Individual results are represented by circles, and acute and convalescent pairs are connected by lines. Black triangles represent two ZIKV-positive cases by the composite reference without IgM against ZIKV. The dashed line represents the cut-off value (0.214) for the ZIKV MAC-ELISA calculated by the receiver operating characteristic analysis.

The ZIKV IgM antibody results for paired samples are shown in Figure 1. As expected, the detection rate of antibody against ZIKV was higher in convalescent than acute samples (Figure 1, left panel). According to the composite reference, ZIKV-negative cases did not have IgM against ZIKV (Figure 1, central panel), and ZIKV-positive cases were predominantly positive in their convalescent samples (Figure 1, right panel).

**Figure 2:**
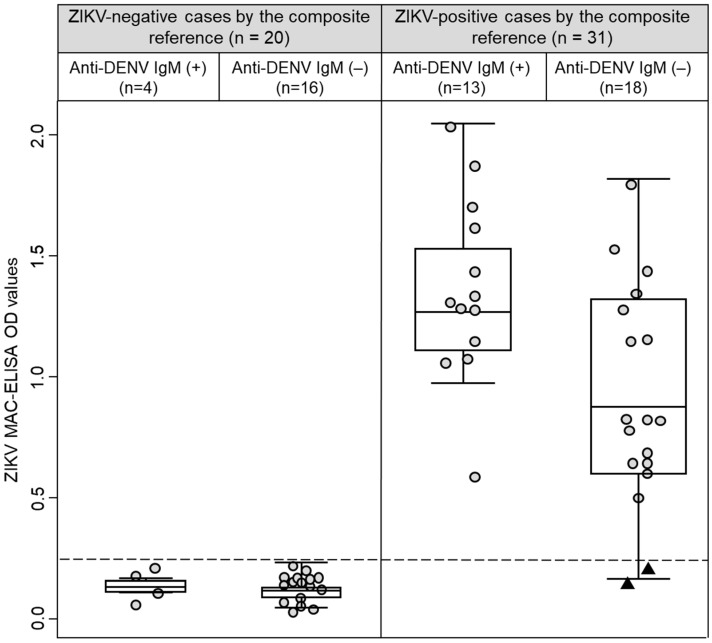
Box-and-whisker plot graphing the ZIKV MAC-ELISA optical density values stratified by anti-DENV IgM results and the composite reference. Convalescent sample’s results are represented by circles. Black triangles represent two cases that were classified as ZIKV-positive by the composite reference and had negative results for anti-ZIKV and anti-DENV IgM antibodies. The dashed line represents the cut-off value (0.214) for the ZIKV MAC-ELISA calculated by ROC analysis.

Based on the composite reference, all 29 participants that had ZIKV IgM antibody in their convalescent samples (Table 1) were also classified as ZIKV-positive cases. Interestingly, two ZIKV-positive cases did not have IgM against ZIKV in their acute and convalescent samples (Figure 1, black triangles at the bottom of the right panel). Of these two cases, one had a positive real-time qRT-PCR result (Participant 41, Ct value = 23.8) and had negative results by MNT, and the other case was seroconverted by MNT (Participant 25, (negative to > 1/640)). Remarkably, both cases were DENV IgM antibody negatives and had multitypic immunity to DENV (Figure 2, black triangles at the bottom of the right panel). Among ZIKV-negative cases, the OD values in the ZIKV MAC-ELISA were comparable between cases with positive or negative results for DENV IgM antibodies (*p* = 0.887). However, among ZIKV-positive cases, the median OD for ZIKV MAC-ELISA was higher in cases with DENV IgM antibody compared to cases without DENV IgM antibody (1.343 vs. 0.842, *p* = 0.010) (Figure 2). In an exploratory analysis, we did not observe any differences in the sample collection time (*p* = 0.470) between these ZIKV-positive cases subgroups. Additionally, it is important to mention that 13 participants had -DENV and ZIKV IgM antibodies and were also classified as ZIKV-positive cases by the composite reference.

The ZIKV MAC-ELISA performance was estimated using convalescent samples due to the low evidence of ZIKV IgM antibodies in acute samples. The overall sensitivity and specificity were 93.5% and 100.0%, respectively (Table 2). Restricting the analysis according to DENV IgM and neutralizing antibodies, the ZIKV MAC-ELISA displayed comparable performance despite the results for DENV testing (Table 2).

### 3.2. Assessment of the ZIKV PRNT

We detected ZIKV neutralizing antibody in convalescent samples of 31 (60.8%) participants (Table 1). We detected two discrepancies between PRNT results and the composite reference; one patient was ZIKV PRNT-negative but positive by the composite reference (Participant 41: Ct value by the ZIKV real-time qRT-PCR = 23.8, and ZIKV-negative by MNT), and the other patient was ZIKV PRNT-positive (PRNT titer: 1/270) but negative by the composite reference (Patient ID: 18, ZIKV-negative by real-time qRT-PCR and seroconversion).

**Figure 3:**
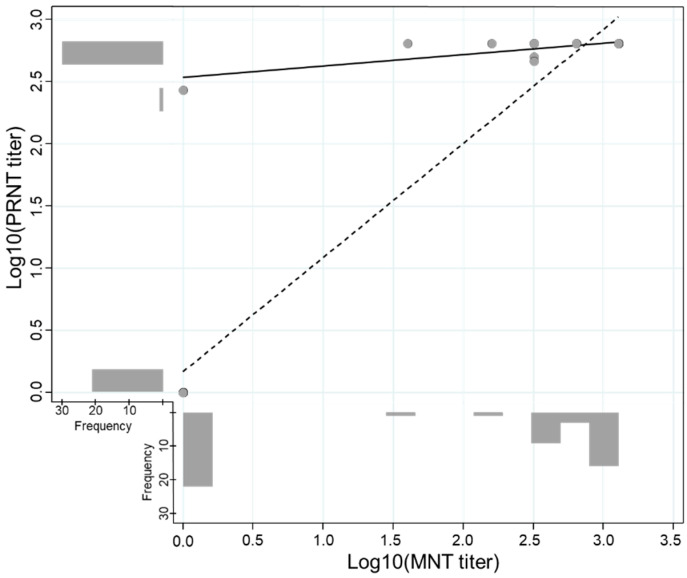
Correlation of log-transformed titers of the Plaque Reduction Neutralization (PRNT) and Microneutralization (MNT) Tests. The scatter plot illustrates the correlation between the log-transformed PRNT and MNT titers. The correlation including and excluding negative results is represented by the dashed line (*n =* 31, Spearman’s Rho = 0.419, *p*-value = 0.019) and solid line (*n =* 51, Spearman’s Rho = 0.901, *p*-value < 0.001), respectively.

The overall sensitivity and specificity for the PRNT detecting the ZIKV type-specific antibody were 96.8% and 95.0%, respectively. The PRNT performance was good and comparable despite the previous DENV infection and days post-onset of symptoms (Table 3). An exploratory analysis conducted to evaluate the agreement between MNT and PRNT in convalescent samples revealed a high kappa value (k = 0.959, 95% CI: 0.880–1.000) and an adequate titer correlation between both methods (Figure 3).

## 4. Discussion

The real-time qRT-PCR and serological assays are complementary and reliable diagnostic tools when used at the right time considering the disease course [29]. The real-time qRT-PCR detects the viral RNA, whereas serological assays are designed to detect antibodies against pathogens beyond the first week after the onset of symptoms [12,30]. In comparison to the real-time qRT-PCR, serological assays such as MAC-ELISA and PRNT do not require expensive supplies and equipment, sophisticated laboratory facilities or highly professional capacity building. These characteristics make serological assays more accessible and useable by resource-limited laboratories. However, the diagnosis of ZIKV infection in dengue-endemic areas using serological assays is doubtful due to the cross reaction between both flaviviruses [14,31,32]. Based on our findings, the diagnostic performance of both serological assays described here was suitable and displayed adequate discriminative power even among patients with a previous DENV infection.

The development and availability of the MAC-ELISA for ZIKV infection screening has greatly expanded the capacity to diagnose ZIKV cases [33]. Overall, our results suggest that the ZIKV MAC-ELISA was a reliable laboratory test even in cases with confirmed multiple DENV infections and could be used as a reference tool in cases with suspected ZIKV infection with the DENV IgM antibody that requires further evaluation. In this study, the MAC-ELISA specificity was 100% for identifying the ZIKV type-specific antibody and it does not seem to be affected by the DENV IgM antibody or neutralizing antibodies elicited by DENV. Despite the sensitivity variation across the evaluated groups, our estimations were comparable to previous in-house and commercial immunoassays described elsewhere [24,33]. Interestingly, the ZIKV MAC-ELISA failed to detect two ZIKV-positive cases (Participant 25 and 41) despite their laboratory-confirmed ZIKV infection by the real-time qRT-PCR or seroconversion by MNT. Overall, the average time elapsed between the sample collection of acute and convalescent serum samples was 18.1 ± 6.1 (mean ± standard deviation) days, but the time between the collection of the acute and the convalescent samples for these two cases was less than the average, placing them below the 5th percentile. Specifically, the time elapsed in one case (Participant 41) was 11 days, and in the other (Participant 25) it was 12 days, resulting in a total time of 13 and 14 days after the onset of symptoms, respectively. It is possible that the reduced time elapsed for both sample collections in these two cases was due to the failure of the patient to develop detectable levels of the IgM antibody because of the kinetics of the immune response in the early disease course or to the possibility of the patient being immunocompromised.

PRNT is frequently used to detect and determine the titer neutralizing antibodies and also to distinguish virus infections among related flaviviruses such as ZIKV and DENV [12,13,17,34,35]. The ZIKV PRNT evaluated here displayed adequate performance with sensitivity and specificity values above 93% regardless of previous DENV infection. Interestingly, the sensitivity or specificity decreased in cases with less than 22 days after the onset of symptoms but was comparable over the time. On the other hand, we detected two presumptive inconsistencies in the ZIKV PRNT results. One case (Participant 41) was ZIKV PRNT-negative but real-time qRT-PCR-positive. Negative ZIKV MAC-ELISA and PRNT results for this case and the reduced time elapsed in the sample collection, as described above, support the hypothesis of not enough time for the development of a detectable antibody response. The other case (Participant 18) was classified as the ZIKV-negative case by the composite reference but was ZIKV PRNT-positive with a titer of 1/270. Remarkably, this case displayed a multitypic DENV profile and all Zika cases displayed titers ranging from 1/500 to ≥1/640. The presumptive inconsistency could be explained by a recent DENV infection that elicited cross-reactive antibodies to ZIKV that were detected by the ZIKV PRNT.

Our results are subject to multiple limitations. The ZIKV in-house antigen used in the MAC-ELISA was an inactivated viral native particle without quantification. Therefore, the results may vary according to the production batches. The IgM antibody cross-reactivity elicited by ZIKV and DENV infection have already been documented at different rates [14,15,16]. In this study, the high cross-reactivity detected in the screening for DENV and ZIKV IgM antibodies among ZIKV-real-time-qRT-PCR confirmed that cases could be explained by the use of the native antigen. However, the mechanism of cross-reactivity was not evaluated in this study. The small sample size in the DENV naïve and monotypic groups generated wider confidence intervals in the sensitivity and specificity estimation. It is important to mention that this study was conducted using samples from persons with compatible symptoms of arbovirus disease who live in dengue-endemic areas; therefore, the low probability of enrolling persons without previous DENV infections resulted in the small sample size. Moreover, all of the analyses were conducted considering DENV infection as profiles; consequently, results could not be interpreted in terms of DENV serotypes. Additionally, it is important to mention that the serological tests described here may have limited diagnostic utility during the first days of illness since antibody production is low. Thus, molecular testing is strongly recommended within the first days of illness. Finally, the use of different reference standards or the use of a unique imperfect test as a reference could lead to biased evaluations [25,26,27]. In this study, we constructed a composite reference standard using the results of one molecular and one serological assay as performed in a previous ZIKV study [24]. This statistical approach combines multiple imperfect reference standards in order to reduce the bias in the absence of a perfect standard, though this method does not eliminate the latent misclassification associated to each imperfect reference standards [36,37]. However, the high correlation among MNT and PRNT titers and the high agreement observed when the data were dichotomously analyzed suggested that our results were not affected by the inclusion of the MNT in the composite reference standard.

## 5. Conclusions

In conclusion, both the in-house MAC-ELISA and PRNT were reliable for the diagnosis of ZIKV infection despite previous DENV infections. Collectively, our results support the use of these serological tests as convenient and reliable laboratory tools for the diagnosis of ZIKV infection in resource-limited settings located in endemic DENV areas with no access to commercial serologic tests.

## Figures and Tables

**Table 1:** Results for both Zika and Dengue virus diagnostic testing (*n =* 51).

Laboratory Assay	*n* (%)
ZIKV-RNA ^1^	
Positive	27 (52.9)
Negative	24 (47.1)
ZIKV seroconversion by MNT ^2^	
Yes	30 (58.8)
No	21 (41.2)
ZIKV MAC-ELISA ^3^	
Positive	29 (56.9)
Negative	22 (43.1)
Neutralization antibodies for ZIKV by PRNT ^3^	
Yes	31 (60.8)
No	20 (39.2)
DENV MAC-ELISA ^3^	
Positive	17 (33.3)
Negative	34 (66.7)
Neutralization antibodies for DENV by PRNT ^3^	
Naïve	2 (3.9)
Monotypic	4 (7.8)
Multitypic	45 (88.3)
ZIKV infection by the composite reference ^4^	
Yes	31 (60.8)
No	20 (39.2)

^1^ ZIKV-RNA was tested in acute serum samples. ^2^ The seroconversion was detected using the paired acute and convalescent serum sample. ^3^ Results in convalescent serum samples. ^4^ Participants were classified as ZIKV-positive cases by the composite reference if ZIKV-RNA was detected in the acute sample, displayed seroconversion in paired serum samples by ZIKV MNT, or both.

**Table 2:** MAC-ELISA performance for ZIKV diagnosis.

Groups	*n*/N	%	95% CI
Overall (*n =* 51)			
Sensitivity	29/31	93.5	78.6–99.2
Specificity	20/20	100.0	83.2–100.0
Anti-DENV IgM			
Positive (*n =* 17)			
Sensitivity	13/13	100.0	75.3–100.0
Specificity	4/4	100.0	39.8–100.0
Negative (*n =* 34)			
Sensitivity	16/18	88.9	65.3–98.6
Specificity	16/16	100.0	79.4–100.0
DENV neutralizing antibodies			
DENV naive (*n =* 2)			
Sensitivity	1/1	100.0	2.5–100.0
Specificity	1/1	100.0	2.5–100.0
DENV monotypic (*n =* 4)			
Sensitivity	0/0	N.A.	N.A.
Specificity	4/4	100.0	39.8–100.0
DENV multitypic (*n =* 45)			
Sensitivity	28/30	93.3	77.9–99.2
Specificity	15/15	100.0	78.2–100.0

Note: Sensitivity and specificity were estimated by considering true results (*n*; positive or negative) over the total (N) of positives and negatives according to the reference, respectively. Not applicable (N.A.).

**Table 3:** PRNT performance for ZIKV diagnosis.

Groups	*n*/N	%	95% CI
Overall (*n =* 51)			
Sensitivity	30/31	96.8	83.3–99.9
Specificity	19/20	95.0	75.1–99.9
DENV neutralizing antibodies			
DENV naive (*n =* 2)			
Sensitivity	1/1	100.0	2.5–100.0
Specificity	1/1	100.0	2.5–100.0
DENV monotypic (*n =* 4)			
Sensitivity	0/0	N.A.	N.A.
Specificity	4/4	100.0	39.8–100.0
DENV multitypic (*n =* 45)			
Sensitivity	29/30	96.7	82.8–99.9
Specificity	14/15	93.3	68.1–99.8
Days post-onset of symptoms			
12–14 (*n =* 9)			
Sensitivity	7/8	87.5	47.3–99.7
Specificity	1/1	100.0	2.5–100.0
15–21 (*n =* 25)			
Sensitivity	13/13	100.0	75.3–100.0
Specificity	11/12	91.7	61.5–99.8
22–28 (*n =* 10)			
Sensitivity	6/6	100.0	54.1–100.0
Specificity	4/4	100.0	39.8–100.0
29–33 (*n =* 7)			
Sensitivity	4/4	100.0	39.8–100.0
Specificity	3/3	100.0	29.2–100.0

Note: Sensitivity and specificity were estimated by considering true results (*n*; positive or negative) over the total (N) of positives and negatives according to the reference, respectively. Not applicable (N.A.).

## Supplementary Materials

The following are available online at https://www.mdpi.com/article/10.3390/diagnostics11091696/s1, Table S1: Cross-reactivity evaluation of the microneutralization test (MNT) and plaque reduction neutralization test (PRNT) using a set of 38 DENV-positive serum samples, Table S2: Receiver operating characteristic analysis and its detailed report of the MAC-ELISA performance for several potential cut-off values.
