# Supplementary material for: Evaluation of Two Serological Assays for Diagnosing Zika Virus Infection
Source: Diagnostics (Basel). 2021 Sep 17;11(9):1696. doi: 10.3390/diagnostics11091696 (PMC8469165; doi:10.3390/diagnostics11091696)
Supplement: Supplementary file 1 [file diagnostics-11-01696-s001.zip › diagnostics-1329640-supplementary.pdf]

Table S1. Cross-reactivity evaluation of the microneutralization test (MNT) and plaque reduction neutralization test (PRNT) using a set of 38 DENV-positive serum samples

	ZIKV MNT and PRNT	
Serum samples positive for:	Positive<br>% (n/N)	Negative<br>%(n/N)
DENV-1	0.0 (0/10)	100.0 (10/10)
DENV-2	0.0 (0/10)	100.0 (10/10)
DENV-3	0.0 (0/10)	100.0 (10/10)
DENV-4	0.0 (0/8)	100.0 (8/8)

Note: DENV infection was confirmed by at least one of the following methods; real-time qRT-PCR, viral isolation + immunofluorescence assay, or seroconversion determined by IgM against DENV considering a titer increase of ≥4-fold.

Table S2. Receiver operating characteristic analysis and its detailed report of the MAC-ELISA performance for several potential cut-off values

Cut-off	Sensitivity (%)	Specificity (%)	Correctly classified (%)
0.173	93.5	85.7	88.5
0.202	93.5	90.5	90.4
0.208	93.5	95.2	94.2
0.214	93.5	100.0	96.2
0.476	90.3	100.0	94.2
